# Dietary Pattern and Nutrients Intake on Chronic Diseases

**DOI:** 10.3390/nu15153399

**Published:** 2023-07-31

**Authors:** Rosa Casas

**Affiliations:** 1 Centro de Investigación Biomédica en Red (CIBEROBN) de Fisiopatología de la Obesidad y Nutrición, Instituto de Salud Carlos III, 28029 Madrid, Spain; rcasas1@recerca.clinic.cat; 2 Department of Internal Medicine, Hospital Clinic, Institut d’Investigació Biomèdica August Pi i Sunyer (IDIBAPS), Villarroel 170, 08036 Barcelona, Spain; 3 Institut de Recerca en Nutrició i Seguretat Alimentaria (INSA-UB), University of Barcelona, 08007 Barcelona, Spain

Non-communicable diseases (NCDs) are the leading causes of mortality and morbidity worldwide, primarily affecting middle-aged men, and imposing a significant global public health burden. According to the World Health Organization (WHO), NCDs are responsible for the majority of deaths and disability globally. Global data reports that CVD directly accounts for 17.9 million deaths annually, followed by cancer (9.3 million), chronic respiratory diseases (4.1 million), and diabetes (2.0 million, including diabetes-related kidney disease deaths). The burden of CVD observed across different time periods and countries can be explained by factors such as globalization, industrialization, demographic and socio-economic changes, unhealthy diets, lack of physical activity, exposure to tobacco smoke, and excessive alcohol consumption. It is estimated that 85% of deaths, mainly from coronary heart disease and cerebrovascular diseases, occur in developing countries. NCDs disproportionately affect individuals in low- and middle-income countries, with 86% of premature deaths from NCDs occurring in these regions. The increasing burden of NCDs has made their prevention and management a global priority. NCDs share common etiopathological factors, all of which point to a specific treatment approach: diet.

NCDs are strongly influenced by lifestyle factors, and diet is among the top five risk factors for mortality. Modifying lifestyle risk factors is crucial for preventing cardiovascular events and reducing the associated risk factors for these diseases. Among all lifestyle factors, dietary habits play a key role in prevention. Multiple studies have shown that a well-balanced diet, providing sufficient essential nutrients in an accessible, safe, and varied manner, not only prevents malnutrition but also reduces the risk of developing NCDs. Therefore, promoting healthy lifestyles is necessary to combat NCDs and, consequently, reduce the financial burden on healthcare systems.

With this purpose in mind, the objective of this Special Issue was to identify and assess healthy dietary patterns (DPs) and specific nutrients in the prevention and management of NCDs. Additionally, the aim was to identify gaps in knowledge that can help uncover the underlying mechanisms involved in this protective effect.

The Special Issue includes six articles, including a meta-analysis and a narrative review. The research topics covered include the role of dietary patterns in Hepatitis C virus (HCV) infection, the effects of a traditional Mediterranean Diet (MedDiet) on otitis media with effusion, the impact of MedDiet on the gut microbiota of individuals infected with human immunodeficiency virus (HIV-1), the clinical implications of MedDiet before and after bariatric surgery, the association between immunoglobulin A (IgA) levels and the prevention of influenza infections, and the relationship between ultra-processed foods and the nutritional dietary profile.

Ojeda-Granados et al. [1] demonstrated that modifying unhealthy dietary patterns and targeting HCV-interacting nutrients could be part of a nutritional management strategy to prevent further liver damage in individuals with a chronic HCV infection (CHC). They assessed the nutritional profiles of Mexican patients with CHC and spontaneous clearance (SC) in the context of APOE alleles and their correlation with HCV-related variables. The authors reported that a high adherence to a fish-rich dietary pattern was associated with a higher frequency of CHC individuals consuming polyunsaturated fatty acids (PUFAs) and having a low viral load. However, it was also associated with a lower frequency of CHC individuals consuming dietary fiber. On the other hand, Gonda et al. [2] emphasized the benefits of consuming vegetables abundant in polyphenols on a daily basis. The authors reported that patients who regularly consumed Okinawan vegetables (200–300 g/day for ≥300 days/year) had higher levels of immunoglobulins (IgA, IgG, and IgM) compared to those who did not. Therefore, a higher intake of vegetables was associated with higher IgA levels and an increased probability of developing immunity against influenza.

In a study focused on the MedDiet pattern, Calatayud-Sáez et al. [3] provided evidence that otitis media with effusion, a common condition in pediatric primary care, could be prevented and treated with the application of a traditional MedDiet. After one year, 85% of boys and girls between 18 months and 5 years old who were revisited had normalized tympanometry, while the remaining 15% showed decreased symptoms and improved audiometry. Also, in a randomized clinical trial led by Pastor-Ibañez et al. [4], adherence to a supplemented MedDiet was found to positively influence the gut microbiota of individuals infected with HIV-1. The study observed increased abundances of *Bifidobacterium* and improvements in metabolic parameters, immune activation, and Treg function. Finally, Gastaldo et al. [5] contributed a narrative review summarizing the existing research on the clinical impact of a MedDiet before and after bariatric surgery, focusing on its effects on weight loss and improvements in comorbidities. The review suggests that adherence to a Mediterranean Diet may be directly linked to weight loss before or after bariatric surgery. However, more research is needed to ensure the use of the Mediterranean pattern to combat the associated comorbidities, improve micronutrient status, and maintain a healthy body weight in the long term.

Lastly, a meta-analysis conducted by Martini et al. [6] provided high-quality evidence that increased consumption of ultra-processed foods negatively impacts the nutritional quality of diets. Higher intake of ultra-processed foods was associated with increased levels of free sugars, total fats, and saturated fats, as well as decreased levels of fiber, protein, potassium, zinc, magnesium, and vitamins A, C, D, E, B12, and niacin.

Based on the results presented in this Special Issue focused on chronic diseases, adopting healthy dietary patterns has been associated with improved population health. Therefore, a shift towards healthy dietary patterns may contribute to reducing the current unsustainable high levels of NCDs such as CVD, obesity, diabetes, and cancer. Further studies are needed to clarify the cause–effect relationship between dietary patterns, health management, and NCDs, and to provide recommendations for preventing chronic metabolic diseases in middle-aged and elderly populations.
